# Circulating Cell-Free Microbial DNA Signatures and Plasma Soluble CD14 Level Are Associated with Clinical Outcomes of Anti-PD-1 Therapy in Advanced Melanoma Patients

**DOI:** 10.3390/ijms252312982

**Published:** 2024-12-03

**Authors:** Bernadeta Drymel, Katarzyna Tomela, Łukasz Galus, Agnieszka Olejnik-Schmidt, Jacek Mackiewicz, Mariusz Kaczmarek, Andrzej Mackiewicz, Marcin Schmidt

**Affiliations:** 1 Department of Biotechnology and Food Microbiology, Poznań University of Life Sciences, 60-627 Poznań, Poland; agnieszka.olejnik-schmidt@up.poznan.pl; 2 Department of Cancer Immunology, Poznań University of Medical Sciences, 61-866 Poznań, Poland; ktomela@gmail.com (K.T.); mariusz.kaczmarek@wco.pl (M.K.); andrzej.mackiewicz@wco.pl (A.M.); 3 Department of Medical and Experimental Oncology, Institute of Oncology, Poznań University of Medical Sciences, 60-355 Poznań, Poland; lukasz_galus@wp.pl (Ł.G.); jmackiewicz@ump.edu.pl (J.M.); 4 Department of Cancer Diagnostics and Immunology, Greater Poland Cancer Centre, 61-866 Poznań, Poland

**Keywords:** circulating cell-free microbial DNA, plasma soluble CD14, advanced melanoma, immune checkpoint inhibitors

## Abstract

An accumulating number of studies suggest the potential of circulating cell-free microbial DNA (cfmDNA) as a non-invasive biomarker in various diseases, including cancers. However, its value in the prediction or prognosis of clinical outcomes of immune checkpoint inhibitors (ICIs) is poorly explored. The circulating cfmDNA pool may also reflect the translocation of various microbial ligands to the circulatory system and may be associated with the increased release of soluble CD14 (sCD14) by myeloid cells. In the present study, blood samples were collected from advanced melanoma patients (*n* = 66) before and during the anti-PD-1 therapy (approximately 3 and 12 months after the start). Then, V3-V4 16S rRNA gene sequencing was performed to analyze the circulating cfmDNA extracted from plasma samples. Moreover, the concentration of plasma sCD14 was measured using ELISA. As a result, the differences in the circulating cfmDNA profiles were found between patients with favorable and unfavorable clinical outcomes of the anti-PD-1 and baseline signatures correlated with progression-free survival and overall survival. Moreover, there was a higher concentration of plasma sCD14 in patients with unfavorable clinical outcomes. High baseline sCD14 level and its increase during the therapy prognosticated worse survival outcomes. Taken together, this preliminary study indicates the potential of circulating cfmDNA signatures and plasma sCD14 levels as biomarkers of clinical outcomes of ICIs.

## 1. Introduction

Melanoma is an aggressive form of skin cancer that accounts for the vast majority of skin-cancer-related deaths [1]. Moreover, global reports demonstrated an increasing trend in the number of new melanoma cases and deaths [2,3]. Therefore, effective strategies for detection, treatment, and monitoring are inevitable to reduce melanoma-associated mortality rates.

One of the treatment approaches that demonstrates clinical activity in various malignancies, including advanced melanoma, is immunotherapy with the use of immune checkpoint inhibitors (ICIs), such as inhibitors of cytotoxic T lymphocyte antigen-4 (CTLA-4), programmed death 1 (PD-1), and its ligand (PD-L1). ICIs restore anti-tumor immune responses through the disruption of co-inhibitory T-cell signaling. Unfortunately, a subset of patients fails to respond or initially respond but eventually develop the progression of the disease. Various tumor-intrinsic and -extrinsic factors may be involved in the mechanism of response and resistance to the therapy [4,5]. For instance, an accumulating number of studies suggests the association of the gut microbiota with clinical outcomes of ICI therapy [6,7,8,9,10]. However, one of the limitations of the gut microbiota analysis is the need for stool sample collection, which may be challenging from the patient’s perspective due to its undesirable nature, and a subset of them may refuse to participate in such studies [11]. Moreover, physical impairments may also interfere with stool collection for gut microbiota analysis. Currently, it is not clear whether microbial signatures from other human specimens may also have the potential to become biomarkers of clinical outcomes of ICI therapy.

In the present study, the analysis of circulating cell-free microbial DNA (cfmDNA) composition in blood plasma samples derived from advanced melanoma patients undergoing anti-PD-1 therapy was performed. Circulating cfmDNA comprises short fragments of DNA, which may represent microbial sequences (mostly bacterial in origin) of whole-body microbiotas. However, its source and potential functions have not been well characterized. According to recent findings, circulating cfmDNA is considered a potential non-invasive diagnostic biomarker in various diseases, including cancers [12]. For instance, Huang et al. (2018) found a distinct composition of circulating cfmDNA between healthy females and early-onset breast cancer (EOBC) patients with severe symptoms [13]. Interestingly, an EOBC patient whose circulating cfmDNA profile was similar to those observed in healthy individuals has been living a healthy life since surgery in 2007 (reported in 2017). On the other, Zozaya-Valdés et al. (2021) did not find remarkable differences in the circulating cfmDNA between advanced melanoma patients and healthy controls [14]. The association between the circulating cfmDNA signatures and clinical outcomes of ICIs has also been poorly explored. To date, Ouaknine Krief et al. (2019) reported the link between baseline plasma microbiome profile and clinical outcomes of the anti-PD-1 therapy in a cohort with advanced non-small-cell lung cancer (NSCLC) [15]. In the present study, differences in the circulating cfmDNA profiles were found between patients with favorable and unfavorable clinical outcomes, which were the most evident early after the start of anti-PD-1 therapy. Moreover, baseline signatures correlated with survival outcomes. It is also worth noting that circulating cfmDNA composition has changed over the study period regardless of clinical outcome. However, in patients with unfavorable ones, these alterations occurred early after anti-PD-1 initiation, while in those with favorable, at a later stage of treatment.

Furthermore, the soluble CD14 (sCD14) concentration was measured in plasma samples. CD14 is a glycoprotein present in a membrane-bound form (mCD14) on the surface of myeloid cells, such as macrophages, monocytes, neutrophils, and dendritic cells, or in soluble form (sCD14) in the blood and extravascular fluids, such as cerebrospinal fluid (CSF) or synovia [16,17]. CD14 expression was also found in non-immune cells, such as enterocytes, hepatocytes, and pancreatic islet beta cells [18]. Soluble CD14 may be secreted through the proteolytic breakdown of mCD14 or released from intracellular stores [19]. The best-recognized function of CD14 is its ability to bind a wide range of pathogen- and damage-associated molecular patterns (PAMPs and DAMPs, respectively) and act as a co-receptor of Toll-like receptor (TLR) 4 to activate innate immune responses. It was demonstrated that sCD14 may act as an inflammatory co-ligand and stimulate mCD14-negative cells to respond to bacteria. A study on a murine experimental meningitis model revealed that simultaneous intracerebral inoculation of recombinant mouse sCD14 and *Streptococcus pneumoniae* enhanced the release of proinflammatory cytokines, such as interleukin 6 (IL-6) and tumor necrosis factor (TNF) into the CSF and stimulated subarachnoid bacterial growth and/or affected bacterial clearance but did not influence leukocyte infiltration or their inflammatory responsiveness [16]. These data suggest sCD14-dependent response to bacterial stimuli in brain cells. Additionally, an accumulating number of studies suggest an involvement of CD14 signaling in the regulation of various biological processes, such as clearance of apoptotic cells and induction of immunostimulatory or immunosuppressive responses in barrier tissues [19]. CD14 was found to be associated with various infectious, metabolic, cardiovascular, and autoimmune diseases and cancers [18]. In the present study, an increased concentration of plasma sCD14 was found in patients with unfavorable clinical outcomes at baseline and early after the start of the anti-PD-1 therapy. Moreover, its high baseline level correlated with worse survival outcomes. An increase in the concentration of sCD14 at the beginning of treatment was a predictor of poor clinical benefits and prognosticated an increased risk of disease progression and death in the study cohort. Interestingly, trends in sCD14 concentration corresponded with circulating cfmDNA composition and diversity in patients who did not respond to the anti-PD-1 therapy.

Taken together, these findings demonstrate the potential of circulating cfmDNA signatures and plasma sCD14 levels as biomarkers of clinical outcomes of ICIs. To our knowledge, this is the first study demonstrating such an association in advanced melanoma patients undergoing anti-PD-1 therapy.

## 2. Results

### 2.1. Cohort Characteristics

In total, 66 patients with unresectable stage III or stage IV cutaneous melanoma enrolled in the treatment with the anti-PD-1 therapy were recruited in the present study. The analysis of baseline sociodemographic and clinical characteristics did not reveal any statistically significant differences in terms of age, sex, distribution of the metastatic stage, and V600E/K mutation in the *BRAF* gene between study subgroups (*p*-value > 0.05, Table 1). However, the median serum lactate dehydrogenase (LDH) level was significantly higher in patients with unfavorable clinical outcomes compared to those with favorable ones (responders (R) vs. non-responders (NR) and patients who clinically benefited from the anti-PD-1 therapy (CB) vs. those with no clinical benefit (NB), *p*-value < 0.05, Table 1). Moreover, there were also statistically significant differences in the normal (≤250 U/L) and elevated (>250 U/L) serum LDH level distribution between CB and NB subgroups (*p*-value = 0.014, Table 1). In detail, the concentration of serum LDH was in a normal range in 79% of CB and 48% of NB. The baseline characteristics of the study cohort are shown in Table 1.

### 2.2. Taxonomic Profile of the Circulating cfmDNA at the Phylum Level

**Figure 1:**
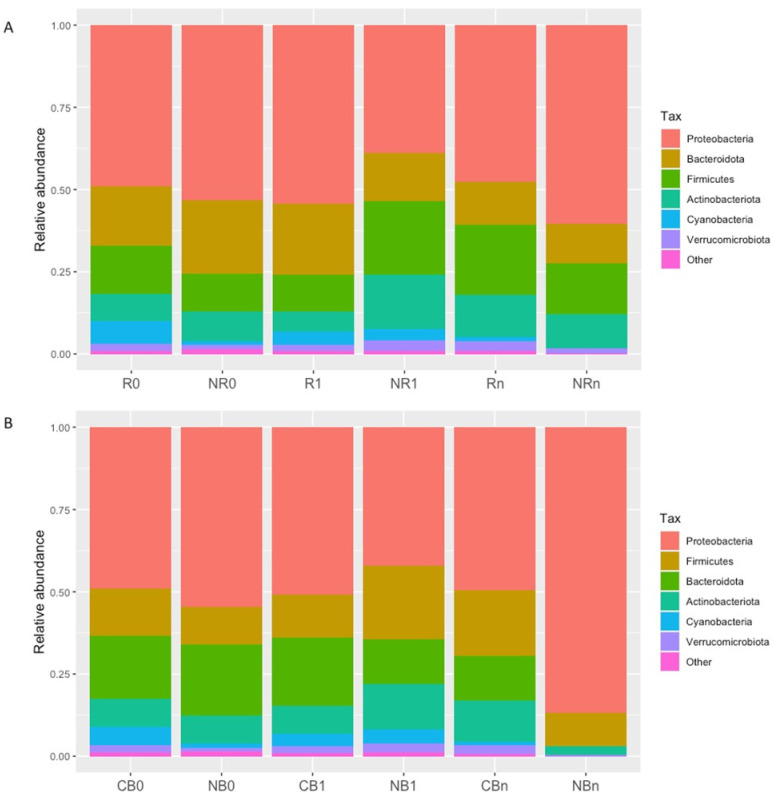
Taxonomic profile of the circulating cell-free microbial DNA (cfmDNA) at the phylum level in advanced melanoma patients receiving the anti-PD-1 therapy, (**A**) classified as responders (R) and non-responders (NR) or (**B**) as patients with clinical benefit (CB) and patients with no clinical benefit (NB) according to the clinical outcome to the ICI therapy, before the anti-PD-1 therapy commencement at T_0_ (0) and during therapy at T_1_ and T_n_ (1 and n, respectively). Circulating cfmDNA was dominated by phyla *Proteobacteria*, *Bacteroidota*, *Firmicutes*, and *Actinobacteriota* in all study subgroups.

The primary goal of our study was to analyze the composition of the circulating cfmDNA. Several reports suggested its potential to become a valuable biomarker in oncology [12]; however, this aspect is poorly explored in cancer patients undergoing ICI therapy. Here, taxonomic profiling at the phylum level revealed that circulating cfmDNA was dominated by the *Proteobacteria* phylum, followed by phyla *Bacteroidota*, *Firmicutes*, and *Actinobacteriota* in all study subgroups (Figure 1).

**Figure 2:**
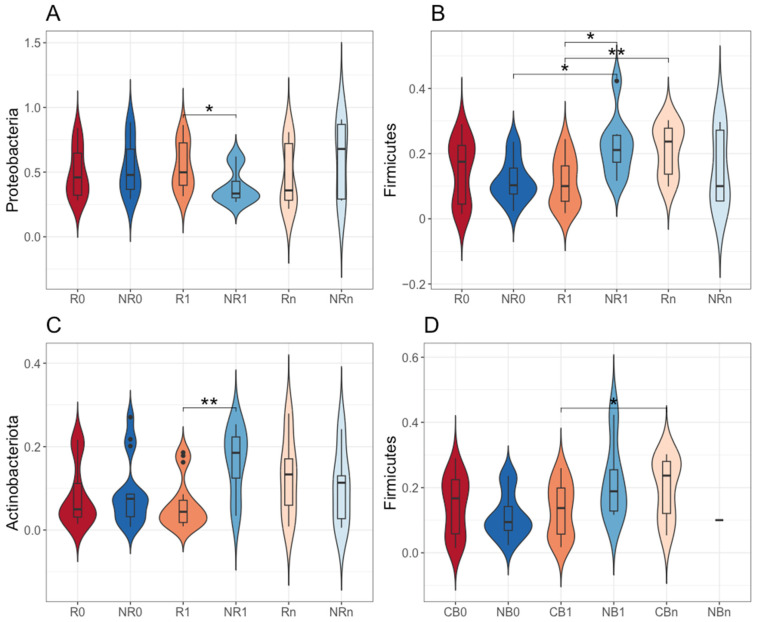
The comparison of the relative abundance of phyla: (**A**) *Proteobacteria*, (**B**,**D**) *Firmicutes*, and (**C**) *Actinobacteriota* in the circulating cell-free microbial DNA (cfmDNA) between advanced melanoma patients receiving the anti-PD-1 therapy, (**A**–**C**) classified as responders (R) and non-responders (NR) or (**D**) as patients with clinical benefit (CB) and patients with no clinical benefit (NB) according to the clinical outcome to the ICI therapy, before the anti-PD-1 therapy commencement at T_0_ (0) and during therapy at T_1_ and T_n_ (1 and n, respectively). The *p*-values describing the statistical significance of the differences in the relative abundance of particular phyla between study subgroups were calculated with the *t*-test. The *p*-values ≤ 0.05 were regarded as significant (*: *p*-values ≤ 0.05, **: *p*-values ≤ 0.01). The analysis indicated a higher relative abundance of *Proteobacteria* phylum and a lower relative abundance of *Firmicutes* and *Actinobacteriota* phyla in the circulating cfmDNA of R as compared to NR at T_1_ and an increase in the relative abundance of *Firmicutes* phylum during treatment regardless of clinical outcome.

However, there were statistically significant differences in the relative abundance of particular phyla between R vs. NR and CB vs. NB subgroups. At T_1_, there was a higher relative abundance of *Proteobacteria* phylum (54% vs. 39%) and a lower relative abundance of *Firmicutes* and *Actinobacteriota* phyla (11% vs. 22% and 6% vs. 17%, respectively) in the circulating cfmDNA of R compared to NR (*p*-value ≤ 0.05, R1 vs. NR1, Figure 2A–C). Moreover, the relative abundance of *Firmicutes* phylum in the circulating cfmDNA increased during treatment regardless of clinical outcome. In detail, in NR, it increased at T_1_, being significantly higher than at T_0_ (*p*-value ≤ 0.05, NR0 vs. NR1, 12% vs. 22%, Figure 2B), while in R and CB, it increased at T_n_, being significantly higher than at T_1_ (*p*-value ≤ 0.05, R1 vs. Rn and CB1 vs. CBn, 11% vs. 21% and 13% vs. 20%, respectively, Figure 2B,D). Other statistically significant differences in the relative abundance of particular phyla in the circulating cfmDNA between study subgroups have not been described here as they referred to noncorresponding subgroups, i.e., subgroups with different clinical outcomes to the anti-PD-1 therapy at different collection time points.

Taken together, although the taxonomic profile of the circulating cfmDNA at the phylum level was comparable between study subgroups (Figure 1), there were also statistically significant differences in the relative abundance of particular phyla between patients with distinct clinical outcomes (the most noticeable early after the start of the anti-PD-1 therapy at T_1_, Figure 2). Moreover, an increase in the relative abundance of *Firmicutes* phylum was found in non-responders at T_1_, while in those who benefited from the immunotherapy, it was found at T_n_ (Figure 2B,D). These findings suggest that alterations in the circulating cfmDNA composition may reflect some unfavorable changes within the immune system and/or body barriers that occurred early after the start of the anti-PD-1 therapy in patients who did not benefit from treatment.

### 2.3. Bacterial ASV Alpha Diversity of the Circulating cfmDNA

**Figure 3:**
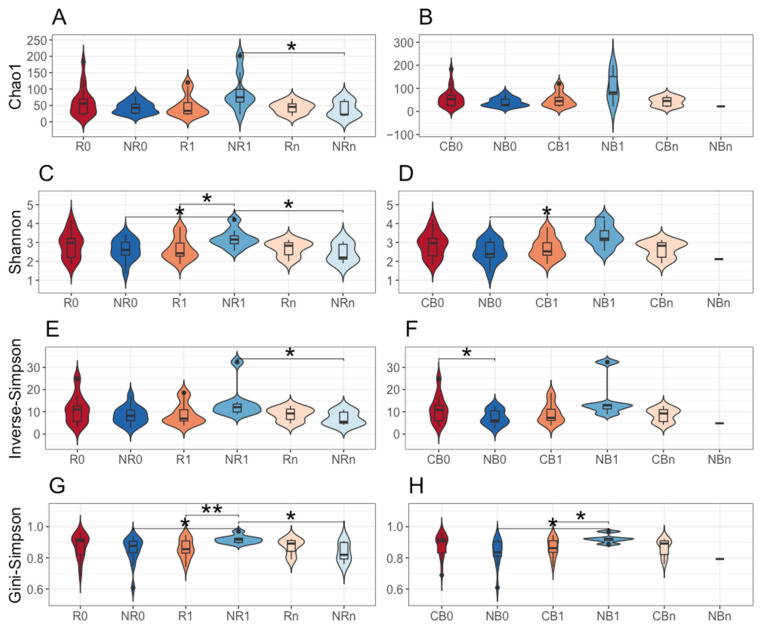
The alpha diversity of circulating cell-free microbial DNA (cfmDNA) was measured using: (**A**,**B**) Chao1, (**C**,**D**) Shannon, (**E**,**F**) inverse Simpson, and (**G**,**H**) Gini–Simpson indices. Comparison between advanced melanoma patients receiving the anti-PD-1 therapy (**A**,**C**,**E**,**G**) classified as responders (R) and non-responders (NR) or (**B**,**D**,**F**,**H**) as patients with clinical benefit (CB) and patients with no clinical benefit (NB) according to the clinical outcome to the ICI therapy, before the anti-PD-1 therapy commencement at T_0_ (0) and during therapy at T_1_ and T_n_ (1 and n, respectively). The *p*-values describing the statistical significance of differences in the circulating cfmDNA alpha diversity between study subgroups were calculated with the *t*-test. The *p*-values ≤ 0.05 were regarded as significant (*: *p*-values ≤ 0.05, **: *p*-values ≤ 0.01). The analysis indicated increased bacterial ASV alpha diversity in patients with unfavorable clinical outcomes at T_1_.

There were also indicated statistically significant differences in the bacterial ASV alpha diversity of circulating cfmDNA between study subgroups. In detail, in NR at T_1_, it was significantly higher than at T_0_ (Shannon and Gini–Simpson indices, *p*-value ≤ 0.05, NR0 vs. NR1, Figure 3C,G) and at T_n_ (Chao1, Shannon, inverse Simpson, and Gini–Simpson indices, *p*-value ≤ 0.05, NR1 vs. NRn, Figure 3A,C,E,G) and also in comparison to R at T_1_ (Shannon and Gini-Simpson indices, *p*-value ≤ 0.05, R1 vs. NR1, Figure 3C,G). Similar trends were found in the comparison between CB and NB subgroups (*p*-value ≤ 0.05, NB0 vs. NB1, CB1 vs. NB1, Figure 3D,H). Interestingly, the diversity (inverse Simpson index) of baseline circulating cfmDNA in NB was significantly lower than in CB (*p*-value ≤ 0.05, CB0 vs. NB0, Figure 3F). Other statistically significant differences in the bacterial ASV alpha diversity of the circulating cfmDNA between study subgroups have not been described here as they referred to noncorresponding subgroups, i.e., subgroups with different clinical outcomes to the anti-PD-1 therapy at different collection time points.

Collectively, bacterial ASV richness and diversity of circulating cfmDNA were increased in patients with unfavorable clinical outcomes at T_1_ compared to other study subgroups (Figure 3), which corresponds with the differences observed in the relative abundance patterns at the phylum level (Figure 2).

### 2.4. The Comparison of the Circulating cfmDNA Signatures Between Patients with Favorable and Unfavorable Clinical Outcomes at Baseline (at T_0_) and During the Anti-PD-1 Therapy (at T_1_ and T_n_)

The differential abundance analysis (DAA) was performed at the genus level to identify differentially abundant taxa in the circulating cfmDNA between patients with favorable and unfavorable clinical outcomes of the anti-PD-1 therapy before and after its commencement.

**Figure 4:**
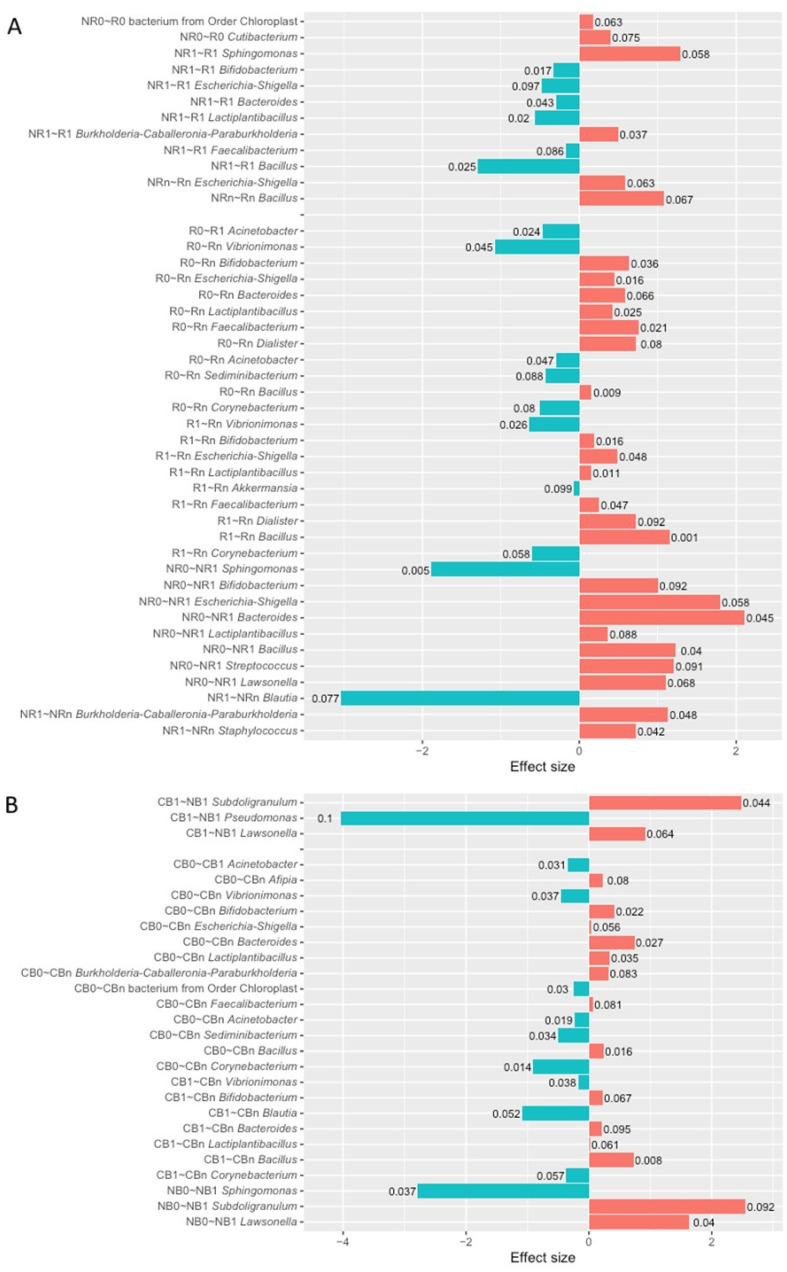
The differential abundance analysis (DAA) was performed using ANOVA-like Differential Expression version 2 (ALDEx2) tool. The differentially abundant taxa in the circulating cell-free microbial DNA (cfmDNA) were identified at the genus level in the comparisons between subgroups of advanced melanoma patients classified as (**A**) responders (R) and non-responders (NR) or (**B**) as patients with clinical benefit (CB) and patients with no clinical benefit (NB) according to the clinical outcome to the ICI therapy, before the anti-PD-1 therapy commencement at T_0_ (0) and during therapy at T_1_ and T_n_ (1 and n, respectively). Moreover, the differentially abundant taxa in comparisons between particular collection time points (T_0_ vs. T_1_, T_0_ vs. T_n_, and T_1_ vs. T_n_) within those subgroups were also indicated with the DAA. The *p*-values describing the statistical significance of the DAA results were placed at the tips of the bars. The figure illustrates only the statistically significant results (the Wilcoxon rank test, *p*-values < 0.1). The direction of changes in the relative abundance of taxa between the two subgroups being compared was assessed based on the effect size value. The first group of the two being compared is considered a reference group, whereas the second one is a tested group (group designations are placed on the left side of the graph at the beginning of the following lines; ‘reference group~tested group’). A positive effect size (red bars) suggests an increase in the relative abundance of a particular taxon in the tested group compared to the reference group, while negative (blue bars)—decrease. Effect size also measures the biological significance of the observed differences (the larger the effect size, the more substantial the difference between subgroups). The names of the differentially abundant taxa are placed on the left side of the graph (next to the subgroup designation). The analysis indicated that circulating cfmDNA signatures were associated with clinical outcomes and have changed during the anti-PD-1 therapy.

At baseline, there were few differences in the circulating cfmDNA composition between study subgroups. In detail, the relative abundance of the *Cutibacterium* genus and bacterium from the *Chloroplast* order was higher in R than NR at T_0_ (*p*-value < 0.1, Figure 4A).

At T_1_, there were identified eight differentially abundant taxa between R and NR subgroups (*p*-value < 0.1, Figure 4A). Among those with the highest statistical significance (*p*-value < 0.05), there was an enrichment in *Burkholderia-Caballeronia-Paraburkholderia* genus in R, while in NR, there was higher relative abundance of genera *Bifidobacterium*, *Bacteroides*, *Lactiplantibacillus*, and *Bacillus.* In the CB vs. NB comparison at T_1_, genera *Subdoligranulum* and *Lawsonella* were more abundant in NB, while the *Pseudomonas* genus was more abundant in CB (*p*-value < 0.1, Figure 4B).

At T_n_, similarly to T_0_, the DAA indicated few differences in the circulating cfmDNA composition between R and NR subgroups. The relative abundance of genera *Escherichia-Shigella* and *Bacillus* was higher in R than NR at T_n_ (*p*-value < 0.1, Figure 4A). It is worth noting that these taxa were enriched in NR at T_1_ (*p*-value < 0.1, Figure 4A). The DAA was not performed for comparison of circulating cfmDNA signatures between CB and NB subgroups at T_n_ due to the low sample size in the NB subgroup.

In sum, the DAA indicated the highest number of differentially abundant genera between patients with distinct clinical outcomes at T_1_ (Figure 4). It should be pointed out that these findings correspond with the differences observed between those subgroups in the relative abundance of particular phyla and bacterial ASV alpha diversity of the circulating cfmDNA (Figure 2 and Figure 3) and may result from activation of some diverse mechanisms in those subgroups that divergently affected anti-PD-1 therapy effects.

### 2.5. Changes in the Circulating cfmDNA Signatures During the Anti-PD-1 Therapy Within Subgroups of Patients with Favorable and Unfavorable Clinical Outcomes (T_0_ vs. T_1_, T_0_ vs. T_n_, and T_1_ vs. T_n_)

The DAA was also performed at the genus level to determine changes in the circulating cfmDNA composition during the anti-PD-1 therapy. The microbial signatures of circulating cfmDNA at particular collection time points were compared (T_0_ vs. T_1_, T_0_ vs. T_n_, and T_1_ vs. T_n_) within subgroups with favorable and unfavorable clinical outcomes.

In patients with favorable clinical outcomes, the composition of the circulating cfmDNA did not change remarkably early after the start of the anti-PD-1 therapy (T_0_ vs. T_1_). In detail, the relative abundance of the *Acinetobacter* genus decreased at T_1_ in the R and CB subgroups (*p*-value < 0.1, Figure 4A,B). More substantial changes were found at T_n_ (T_0_ vs. T_n_ and T_1_ vs. T_n_). In the T_0_ vs. T_n_ comparison, there were indicated 11 and 13 differentially abundant taxa within the R and CB subgroups, respectively (*p*-value < 0.1, Figure 4A,B). The relative abundance of the genera *Vibrionimonas, Acinetobacter*, *Sediminibacterium*, and *Corynebacterium* decreased at T_n_ in the R and CB subgroups (*p*-value < 0.1). In contrast, the relative abundance of the genera *Bifidobacterium, Escherichia-Shigella*, *Bacteroides*, *Lactiplantibacillus*, *Faecalibacterium*, and *Bacillus* increased at T_n_ in those subgroups (*p*-value < 0.1). It should be pointed out that in the CB, but not in the R subgroup, there was a significant decrease in the relative abundance of the bacterium from *Chloroplast* order at T_n_ (*p*-value < 0.05, Figure 4B). This taxon was enriched in the baseline circulating cfmDNA of R vs. NR (Figure 4A). In the T_1_ vs. T_n_ comparison, there were identified 9 and 7 differentially abundant taxa within the R and CB subgroups, respectively (*p*-value < 0.1, Figure 4A,B). The changes in the circulating cfmDNA composition were comparable to those observed in the T_0_ vs. T_n_ comparison. There was also observed a decrease in the relative abundance of the genera *Vibrionimonas* and *Corynebacterium* and an increase in the relative abundance of the genera *Bifidobacterium, Lactiplantibacillus*, and *Bacillus* at T_n_ in the R and CB subgroups (*p*-value < 0.1). Moreover, there was a significant increase in the genera *Escherichia-Shigella* and *Faecalibacterium* in the R subgroup (*p*-value < 0.05).

In patients with unfavorable clinical outcomes, the most substantial differences in the circulating cfmDNA composition were found at T_1_ (T_0_ vs. T_1_ and T_1_ vs. T_n_). In the T_0_ vs. T_1_ comparison, there were identified 8 and 3 differentially abundant taxa within the NR and NB subgroups, respectively (*p*-value < 0.1, Figure 4A,B). There was a decrease in the *Sphingomonas* genus and an increase in the *Lawsonella* genus at T_1_ in the NR and NB subgroups (*p*-value < 0.1). It is worth noting that in the NR, but not in the NB, the relative abundance of the genera *Bifidobacterium*, *Escherichia-Shigella*, *Bacteroides, Lactiplantibacillus*, and *Bacillus* (*p*-value < 0.1) increased. An enrichment in those taxa was also observed in patients with favorable clinical outcomes at T_n_ and NR in comparison to R at T_1_. No statistically significant changes in the circulating cfmDNA were found in the T_0_ vs. T_n_ comparison in the NR subgroup, while in the T_1_ vs. T_n_, there were identified three differentially abundant taxa (*p*-value < 0.1, Figure 4A). In detail, the relative abundance of the genera *Burkholderia-Caballeronia-Paraburkholderia* and *Staphylococcus* increased, while the *Blautia* genus decreased at T_n_. The DAA was not performed for comparison of circulating cfmDNA signatures at T_0_ vs. T_n_ and T_1_ vs. T_n_ within the NB subgroup due to the low sample size in the NB subgroup at T_n_.

Taken together, the relative abundances of particular genera in the circulating cfmDNA have changed during the anti-PD-1 therapy regardless of clinical outcome (Figure 4). However, in patients with favorable clinical outcomes, these alterations were observed at T_n_, whereas in those with unfavorable ones, at T_1_. Moreover, the relative abundances of several genera, including *Bifidobacterium*, *Escherichia-Shigella*, *Bacteroides, Lactiplantibacillus*, and *Bacillus,* have increased during treatment in the R, CB, and NR subgroups. This finding suggests that enrichment of circulating cfmDNA in those genera may have the value of a predictive biomarker and reflect activation of some unfavorable mechanisms affecting immunotherapy efficacy.

### 2.6. The Association Between the Baseline Circulating cfmDNA Signatures and Survival Outcomes

**Figure 5:**
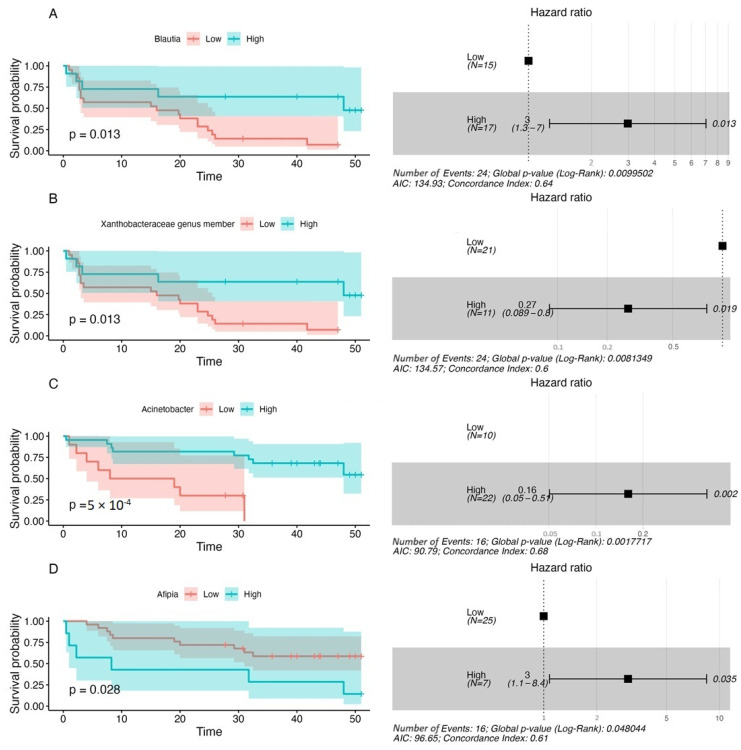
Examples of the most significant associations between the relative abundances of selected taxa in baseline circulating cfmDNA and survival outcomes expressed as progression-free survival—PFS (**A**,**B**) and overall survival—OS (**C**,**D**). PFS and OS rates were plotted with the Kaplan–Meier survival curves (on the left side of the graph), which were compared using the Log-rank (Mantel–Cox) test. The associations between the relative abundance of taxa and survival outcomes were examined with the Cox regression. The *p*-values < 0.1 were regarded as significant. The maximally selected rank statistics were used to determine the relative abundance of microbial taxa (low vs. high). A Cox proportional hazard model was visualized with a forest plot (on the right side of the graph). It shows hazard ratio (HR); HR > 1 indicates an increased risk of disease progression and death, while HR < 1 indicates a decreased risk. The analysis indicated that a high relative abundance of the *Acinetobacter* genus and *Xanthobacteraceae* genus member in the baseline circulating cfmDNA prognosticated improved survival outcomes, while enrichment in the genera *Blautia* and *Afipia* prognosticated poor survival outcomes.

In the present study, the association between the baseline composition of circulating cfmDNA and survival outcomes (progression-free survival—PFS and overall survival—OS) was also investigated. At the genus level, 13 and 7 taxa were associated with OS and PFS, respectively (*p*-value < 0.1, Table S1). A high relative abundance of the *Acinetobacter* genus in the baseline circulating cfmDNA prognosticated decreased risk of disease progression and death (PFS and OS hazard ratio (HR) < 1). Moreover, high relative abundance of the genera *Streptococcus*, *Cutibacterium*, *Roseomonas*, *Vibrionimonas*, and the bacterium from *Chloroplast* order prognosticated reduced risk of death (OS HR < 1), whereas enrichment in the bacterium from *Xanthobacteraceae* family meant decreased risk of disease progression (PFS HR < 1). In contrast, the high relative abundance of the *Kocuria* genus prognosticated increased risk of disease progression and death (PFS and OS HR > 1). Additionally, enrichment in the genera *Afipia*, *Lactiplantibacillus*, *Collinsella*, *Escherichia-Shigella*, *Burkholderia-Caballeronia-Paraburkholderia* prognosticated higher risk of death (OS HR > 1), whereas enrichment in the genera *Blautia* and *Diaphorobacter* meant an increased risk of disease progression (PFS HR > 1). Kaplan–Meier survival curves and a Cox proportional hazard model for taxa most significantly associated with survival outcomes are presented in Figure 5.

### 2.7. The Concentration of Plasma sCD14 and Its Association with Survival Outcomes

**Figure 6:**
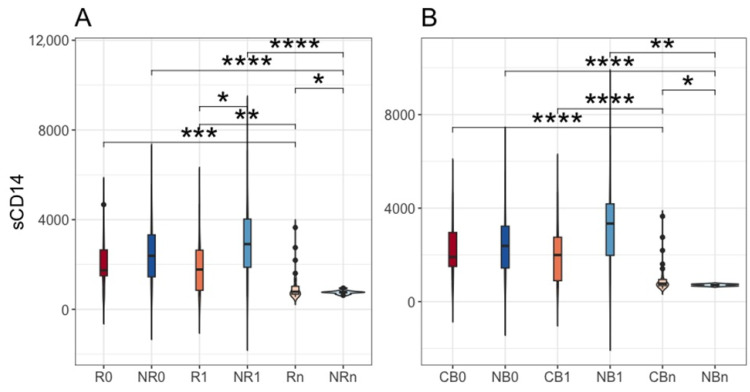
The comparison of plasma soluble CD14 (sCD14) levels between melanoma patients receiving the anti-PD-1 therapy, (**A**) classified as responders (R) and non-responders (NR) or (**B**) as patients with clinical benefit (CB) and patients with no clinical benefit (NB) according to the clinical outcome to the ICI therapy, before the anti-PD-1 therapy commencement at T_0_ (0) and during therapy at T_1_ and T_n_ (1 and n, respectively). The median values for the plasma sCD14 concentrations were (in ng·mL^−1^): 1742.75 (R0), 2385.75 (NR0), 1777.25 (R1), 2910.00 (NR1), 785.05 (Rn), 759.53 (NRn), 1909.00 (CB0), 2385.75 (NB0), 1994.00 (CB1), 3340.25 (NB1), 769.20 (CBn), 708.35 (NBn). The *p*-values describing the statistical significance of the biomarker levels in the study subgroups were calculated with the *t*-test. The *p*-values ≤ 0.05 were found significant (*: *p*-values ≤ 0.05, **: *p*-values ≤ 0.01, ***: *p*-values ≤ 0.001, ****: *p*-values ≤ 0.0001). The values on the y-axis are in ng·mL^−1^. The analysis indicated a significantly higher level of plasma sCD14 in NR than R at T_1_ and in R and CB at T_n_ compared to NR and NB, respectively. Soluble CD14 level decreased at T_n_ regardless of clinical outcome.

The concentration of sCD14 in plasma samples was also measured in the present study. This biomarker is a soluble form of CD14, which is involved in the regulation of various biological processes and was found to be associated with numerous diseases, including tumors [18,19]. Its level was significantly higher in NR than R at T_1_ (*p*-value ≤ 0.05, R1 vs. NR1, median values 1777.25 vs. 2910.00 ng·mL^−1^, respectively, Figure 6A), whereas at T_n_, there was an opposite trend (*p*-value ≤ 0.05, Rn vs. NRn, median values 785.05 vs. 759.53 ng·mL^−1^, respectively, Figure 6A). Notably, these observations correspond with the time points when the changes in the circulating cfmDNA have occurred in R and NR, i.e., at T_n_ and T_1_, respectively (Figure 4A), suggesting that translocation of microbial cells or their parts (cfmDNA or PAMP molecules) to the circulatory system could contribute to the activation of myeloid cells and release of sCD14. It should be pointed out that at T_n_, the concentration of plasma sCD14 in CB was also significantly higher than in NB (*p*-value ≤ 0.05, CBn vs. NBn, median values 769.20 vs. 708.35 ng·mL^−1^, respectively, Figure 6B). However, the difference in the biomarker level between NB and CB at T_1_ did not reach statistical significance (*p*-value > 0.05, CB1 vs. NB1, median values 1994.00 vs. 3340.25 ng·mL^−1^, respectively, Figure 6B). The differences in the circulating cfmDNA signatures between CB1 vs. NB1 and NB0 vs. NB1 were also less evident than in R1 vs. NR1 and NR0 vs. NR1 (Figure 4B). Moreover, the plasma sCD14 level significantly decreased at T_n_ both in R and NR, being significantly lower than at T_0_ and T_1_ within the corresponding subgroups (*p*-value ≤ 0.01, R0 vs. Rn, R1 vs. Rn, NR0 vs. NRn, NR1 vs. NRn, median values written in the description of Figure 6A). Comparable differences were observed in the CB vs. NB comparison (*p*-value ≤ 0.01, CB0 vs. CBn, CB1 vs. CBn, NB0 vs. NBn, NB1 vs. NBn, median values written in the description of Figure 6B). Other statistically significant differences in plasma sCD14 level between study subgroups have not been described here as they referred to noncorresponding subgroups, i.e., subgroups with different clinical outcomes to the anti-PD-1 therapy at different collection time points.

Collectively, there were statistically significant differences in the sCD14 level between patients with distinct clinical outcomes (Figure 6). At T_1_, it was higher in NR than R, while at T_n_, there was an opposite trend (also in CB vs. NB at T_n_). These findings correspond with the changes in the circulating cfmDNA in those subgroups (Figure 4), implying that microbial translocation to the circulatory system could induce sCD14 release. However, non-microbial ligands might also contribute to this response (at T_0_ and T_1_) as the biomarker concentration decreased at T_n_ in both subgroups regardless of clinical outcome (Figure 6).

**Figure 7:**
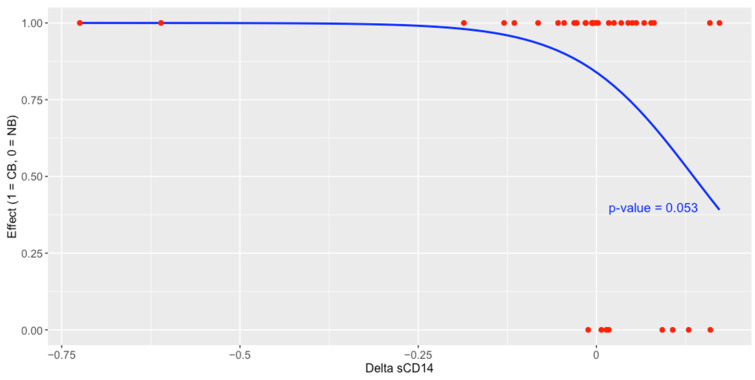
The effect of the change in plasma sCD14 concentration (logarithmic values) between the start of therapy (T_0_) and 3 months after its commencement (T_1_) on the treatment effect expressed as clinical benefit (CB)/no clinical benefit (NB) analyzed with logistic regression. The analysis indicated that an increase in this biomarker was a predictor of the poor effect of ICI therapy.

A logistic regression analysis indicated that an increase in the concentration of plasma sCD14 at T_1_ (as compared to its level at T_0_) was associated with a decreased probability of receiving clinical benefit from the anti-PD-1 therapy (*p*-value = 0.053, Figure 7). 

**Figure 8:**
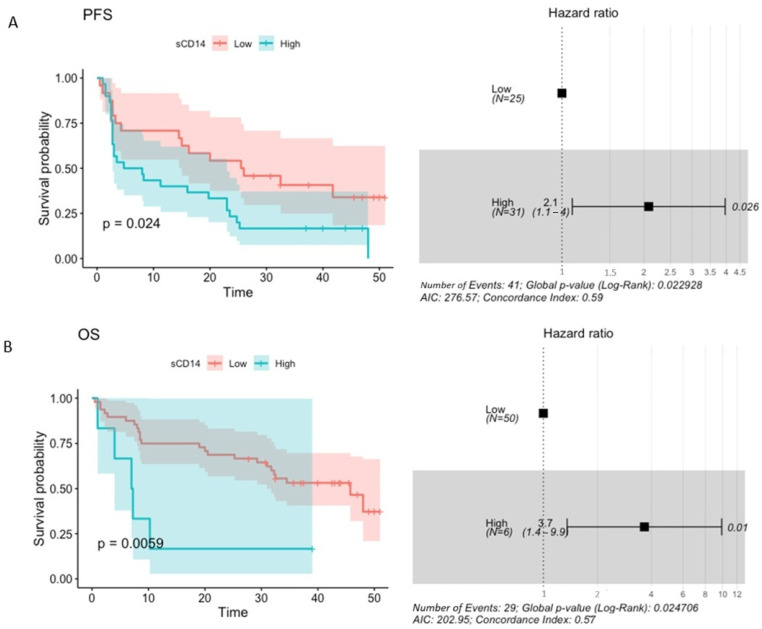
The association between the baseline plasma sCD14 concentration and survival outcomes expressed as progression-free survival—PFS (**A**) and overall survival—OS (**B**). PFS and OS rates were plotted with the Kaplan–Meier survival curves (on the left side of the graph), which were compared using the Log-rank (Mantel–Cox) test. The associations between baseline concentration of plasma sCD14 and survival outcomes were examined with the Cox regression. The *p*-values < 0.1 were regarded as significant. The maximally selected rank statistics were used to determine the plasma sCD14 level (low vs. high). A Cox proportional hazard model was visualized with a forest plot (on the right side of the graph). It shows hazard ratio (HR); HR > 1 indicates an increased risk of disease progression and death, while HR < 1 indicates a decreased risk. This analysis indicated that high baseline sCD14 level prognosticated worse survival outcomes.

**Figure 9:**
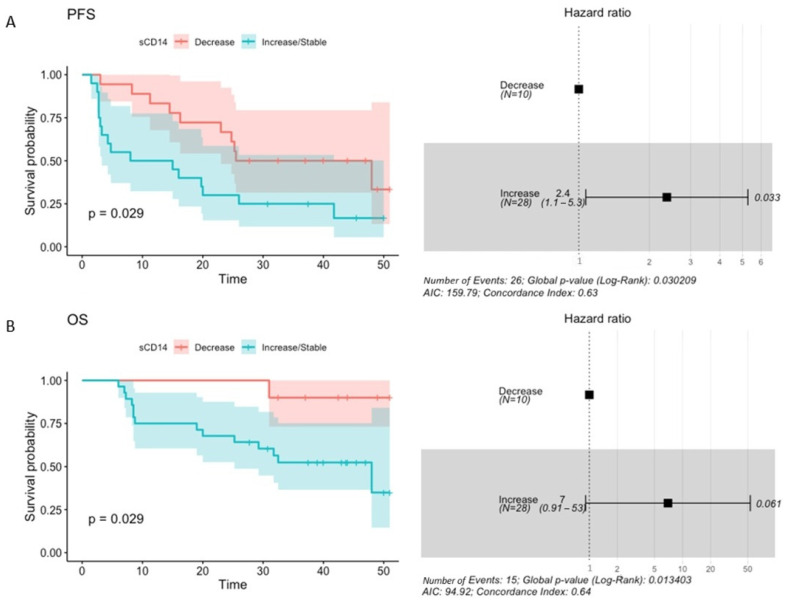
The association between the change (decrease vs. increase/stable) in the plasma sCD14 concentration between the start (T_0_) and 3 months after the start of the anti-PD-1 therapy (T_1_) and survival outcomes expressed as progression-free survival—PFS (**A**) and overall survival—OS (**B**). PFS and OS rates were plotted with the Kaplan–Meier survival curves (on the left side of the graph), which were compared using the Log-rank (Mantel–Cox) test. The associations between changes in concentration of plasma sCD14 and survival outcomes were examined with the Cox regression. The *p*-values < 0.1 were regarded as significant. The maximally selected rank statistics were used to determine the change in plasma sCD14 level (decrease vs. increase/stable). A Cox proportional hazard model was visualized with a forest plot (on the right side of the graph). It shows hazard ratio (HR); HR > 1 indicates an increased risk of disease progression and death, while HR < 1—indicates a decreased risk. This analysis indicated that an increase/stability in sCD14 level at T_1_ prognosticated worse survival outcomes.

Moreover, the baseline level of plasma sCD14 higher than 1992.50 ng·mL^−1^ prognosticated significantly increased risk of disease progression (PFS HR 2.1 (1.1–4), *p*-value = 0.026) and higher than 4187.50 ng·mL^−1^—an increased risk of death (OS HR 3.7 (1.4–9.9), *p*-value = 0.01) in the study cohort (Figure 8A,B). For comparison, median plasma sCD14 concentration in R and CB at T_0_ was 1742.75 ng·mL^−1^ and 1909.00 ng·mL^−1^, respectively. In NR and NB, it was 2385.75 ng·mL^−1^ in both subgroups. Regardless of the clinical outcomes of the anti-PD-1 therapy, the median concentration of plasma sCD14 at T_0_ was comparable to the concentration prognosing shorter PFS and 2 times lower than the concentration prognosing shorter OS in the study cohort. Similarly to the results of the logistic regression analysis, an increase in or stability of plasma sCD14 level between the start (T_0_) and 3 months after the commencement of the anti-PD-1 therapy (T_1_) significantly prognosticated an increased risk of disease progression and death (PFS HR = 2.4 (1.1–5.3), *p*-value = 0.033; OS HR = 7 (0.91–53), *p*-value = 0.061, Figure 9A,B).

In sum, a high baseline concentration of sCD14 and its increase or stability at the beginning of the anti-PD-1 therapy (T_0_ vs. T_1_) were significantly associated with decreased probability of receiving clinical benefit from the immunotherapy (Figure 7) and/or shorter PFS and OS (Figure 8 and Figure 9). These findings suggest the potential of sCD14 to become a predictive and/or prognostic biomarker in cancer patients undergoing anti-PD-1 therapy.

## 3. Discussion

Numerous studies demonstrated the association between the gut microbiota and clinical outcomes of ICIs [6]. However, the link between microbial signatures from other human specimens, such as easily accessible blood samples, and the efficacy of ICI therapy has been poorly explored. Overall, the blood microbiome was found to possess a value of non-invasive biomarker for early cancer detection and survival outcome prediction [20,21]. Moreover, blood microbiome signatures correlated with clinical indices, such as tumor–node–metastasis stage, lymphatic metastasis, tumor diameter, and invasion depth, in gastric cancer patients [22]. An accumulating number of studies suggest the potential of circulating cfmDNA to become a non-invasive biomarker in the detection and monitoring of various diseases [12]. Several studies also characterized circulating cfmDNA signatures in cancer patients. For instance, Huang et al. (2018) suggested its prognostic value in EOBC patients [13]. However, the study cohort comprised a limited number of individuals. Another study indicated higher alpha diversity and enrichment in the *Castellaniella* genus in the circulating cfmDNA of healthy controls compared to advanced melanoma patients [14]. The association between the baseline plasma microbiome profiles and clinical outcomes of the anti-PD-1 therapy in a cohort with advanced NSCLC was also demonstrated [15]; however, the brief description of methods in the manuscript did not ensure whether it was analyzed cfmDNA or microbiota. An enrichment in bacterial DNA from *Peptostreptococcaceae*, *Lewinella*, *Paludibaculum,* and *Holophagae* was associated with response and clinical benefit, while from *Gemmatimonadaceae* with tumor progression and worse OS.

In the present study, the composition of circulating cfmDNA was analyzed in advanced melanoma patients before the start and during the anti-PD-1 therapy to determine whether there were differences in the microbial signatures between patients with favorable and unfavorable clinical outcomes and whether the composition of circulating cfmDNA has changed during the immunotherapy. Overall, *Proteobacteria*, followed by *Bacteroidota*, *Firmicutes*, and *Actinobacteriota,* were the dominant phyla in the circulating cfmDNA of all study subgroups (Figure 1). Comparable abundance patterns were demonstrated in the previous studies [14]. However, there were also observed differences between study subgroups. In detail, at T_1_, the circulating cfmDNA of patients with unfavorable clinical outcomes was characterized by higher bacterial ASV richness and diversity and increased relative abundance of phyla *Firmicutes* and *Actinobacteriota* and decreased relative abundance of *Proteobacteria* phylum compared to patients with favorable ones (Figure 2 and Figure 3). In contrast, lower alpha diversity in the circulating cfmDNA in plasma samples was found in advanced melanoma patients and colorectal cancer patients than in healthy controls [14,23]. Zaidi et al. (2022) found that increased relative abundance of phyla *Actinobacteriota* and *Proteobacteria*, decreased relative abundance of *Firmicutes*, and reduced alpha diversity of the serum microbiome were associated with disease progression to esophageal adenocarcinoma (EAC) [20]. Lower alpha diversity in the serum microbiome was also observed in gastric cancer patients than in healthy controls [22,23]. Furthermore, at T_1_, there were also the most substantial differences in the circulating cfmDNA composition between R vs. NR and CB vs. NB at the genus level (Figure 4). Several of the identified taxa were previously found in the blood specimens of cancer patients, or their enrichment in the gut microbiota was associated with clinical outcomes of ICIs. For instance, genera *Bifidobacterium*, *Lactiplantibacillus*, and *Faecalibacterium*, which are members of the human gut microbiota and possess health-promoting properties [24,25,26], were enriched in the circulating cfmDNA of NR at T_1_ (Figure 4A). The *Bifidobacterium* genus was also more abundant in the serum microbiome of EAC patients compared to those with diseases that may predispose them to cancer development, such as gastroesophageal reflux disease (GERD) [20]. In contrast, enrichment of the gut microbiota in *Bifidobacterium longum* positively correlated with response to ICIs in advanced melanoma and NSCLC patients [27,28]. Similarly, the high relative abundance of *Faecalibacterium* genus in the intestinal microbiota was associated with response and improved survival outcomes in cancer patients undergoing ICI therapy [29,30,31,32,33]. However, in our previous study [9], *Faecalibacterium prausnitzii* was enriched in the stool samples of NR at baseline, which is in line with trends in the circulating cfmDNA (Figure 4A). Moreover, the genus *Escherichia-Shigella* was more abundant in the circulating cfmDNA of NR at T_1_ and R at T_n_ (Figure 4A). *Escherichia* genus and *Escherichia coli* were also enriched in the serum microbiome of EAC patients and metagenome panels that comprised *E. coli* among other microbes, enabled robust discrimination of EAC from GERD patients and prediction of OS in EAC patients [20]. Additionally, *Pseudomonas* and *Sphingomonas* species were found in the circulating cfmDNA of breast cancer patients with severe symptoms [13]; whereas, in another study, these taxa were more abundant in the serum microbiome of healthy controls compared to gastric cancer patients [22]. Moreover, *Sphingomonas* sp. was also enriched in patients with gastric cancer non-lymphatic metastasis vs. those with lymphatic metastasis. In our cohort, the higher relative abundance of genera *Pseudomonas* and *Shingomonas* was found in patients with favorable outcomes at T_1_ and decreased in patients with unfavorable ones during the anti-PD-1 therapy (Figure 4). The *Bacteroides* genus was also enriched in the circulating cfmDNA in NR at T_1_ (Figure 4A). A higher abundance of this genus was reported in the serum microbiome of gastric cancer patients vs. healthy controls and patients with gastric cancer lymphatic metastasis vs. non-lymphatic metastasis [22]. On the other hand, *Bacteroides* sp. was enriched in the serum microbiome of GERD vs. EAC [20]. In the studies on the gut microbiota composition, *Bacteroides* species were associated with either favorable or unfavorable clinical outcomes of ICIs in cancer patients [29,30,31]. In our cohort, *Bacteroides uniformis* was enriched in the baseline stool microbiota of R [9].

Although there were few differences in the baseline circulating cfmDNA composition between patients with favorable and unfavorable clinical outcomes, i.e., enrichment in the genus *Cutibacterium* and bacterium from *Chloroplast* order in R (Figure 4A) and higher alpha diversity (measured by inverse Simpson index) in CB (Figure 3F), baseline signatures significantly correlated with PFS and OS in the study cohort (Table S1, Figure 5). Among those with the highest significance, there were genera *Acinetobacter* and *Xanthobacteraceae* family members that improved survival outcome prognosis, while enrichment in genera *Blautia* and *Afipia* prognosticated worse PFS and OS, respectively (Table S1, Figure 5). Huang et al. (2018) found enrichment in *Acinetobacter* species in healthy individuals and breast cancer patient with near-normal health conditions [13]. In contrast, the *Acinetobacter* genus was also more abundant in the serum microbiome of gastric cancer patients than in healthy controls [22]. The abundance of *Blautia* species in the gut microbiota was also associated with either longer or shorter PFS in advanced melanoma patients undergoing ICI therapy [10,31]. The direction of this relationship was found to be species-dependent. For instance, *Blautia hydrogenotrophica* and *Blautia wexlerae* were enriched in patients with PFS < 12 months and *Blautia schinkii* in those with PFS ≥ 12 months [10]. In another study, *Blautia* species were more abundant in the gut microbiota of non-progressors [8].

Furthermore, the circulating cfmDNA composition has changed during the anti-PD-1 therapy regardless of the clinical outcome. Specifically, there was an increase in the relative abundance of *Firmicutes* phylum, however, in patients with favorable clinical outcomes at T_n_ and in those with unfavorable ones at T_1_ (Figure 2B,D). Consistently, the most substantial differences within the R and CB subgroups were found at T_n_ (T_0_ vs. T_n_ and T_0_ vs. T_n_), while within the NR subgroup, at T_1_ (T_0_ vs. T_1_ and T_1_ vs. T_n_), and were mostly regarded as an enrichment in *Firmicutes* phylum members (Figure 4). Interestingly, there were similar trends in the changes in the circulating cfmDNA composition in those subgroups. In detail, there was an increase in the relative abundance of genera *Bifidobacterium*, *Escherichia-Shigella*, *Bacteroides*, *Lactiplantibacillus*, and *Bacillus* either in R and CB at T_n_ or NR at T_1_. Moreover, those taxa were also enriched in NR at T_1_ (in comparison to R) (Figure 4). The enrichment of circulating cfmDNA in those taxa may suggest activation of some unfavorable mechanisms that affect barriers and enable microbial translocation from various body microbiotas to the circulatory system and may have a predictive biomarker value. On the other hand, there were also observed changes in the relative abundance of distinct taxa in those subgroups, and potentially, those taxa should be considered microbial biomarkers. Further studies are inevitable to improve our understanding of the role of circulating cfmDNA in plasma samples and its potential to become a biomarker in cancer patients undergoing ICI therapy.

Changes in the circulating cfmDNA composition that occurred during the anti-PD-1 therapy (in patients with unfavorable clinical outcomes at T_1_ and in those with favorable ones at T_n_, Figure 2, Figure 3 and Figure 4) also corresponded with trends in the concentration of sCD14 in plasma samples. There was a higher sCD14 level in NR than R at T_1_ and an opposite trend at T_n_, either in R or CB (Figure 6). These findings imply that circulating cfmDNA composition may reflect the general PAMP translocation to the circulatory system from various body microbiotas, which contribute to immune response activation and release of sCD14. However, it is also worth noting that there were not found any statistically significant differences in the fecal zonulin (a biomarker of intestinal permeability [34]) concentration between study subgroups, and circulating cfmDNA showed a distinct composition from the stool microbiome (data not published), suggesting that the gut microbiota, which was found to be associated with clinical outcomes of ICIs in various cancer cohorts [6,7,8,9,10], was not the major source of circulating cfmDNA in our study. One of the other possible sources of microbial DNA is tumor microbiota. There were demonstrated tumor type-specific microbial signatures [35,36]. Moreover, different microbial patterns were associated with distinct host immune responses [36]. Interestingly, there were also differences in the intratumor microbial signatures between metastatic melanoma patients who responded to ICIs vs. those who did not respond [35,36]. On the other hand, other stimuli, apart from microbial ligands, could lead to the upregulated sCD14 release in NR at T_1_ as there was a remarkable decrease in sCD14 level either in patients with favorable or unfavorable clinical outcomes at T_n_ (Figure 6). This finding suggests decreased activation of immune responses in those subgroups at later stages of anti-PD-1 therapy.

Nevertheless, sCD14 was a predictor of poor clinical benefit and survival outcomes in the study cohort. An increase in the concentration of plasma sCD14 at T_1_ was associated with a decreased probability of receiving clinical benefit from the anti-PD-1 therapy (Figure 7). Moreover, high baseline sCD14 level and an increase in or stability of the biomarker level between T_0_ vs. T_1_ were significantly associated with increased risk of disease progression and death (Figure 8 and Figure 9). Interestingly, several previous studies demonstrated the negative correlation between CD14-positive immune cells associated with tumors and survival prognosis in cancer patients [18]. For instance, a large-scale analysis of transcriptome and microarray data demonstrated the correlation between high CD14 expression and poor survival outcomes in colorectal cancer patients [37]. Similarly, intratumoral and peritumoral accumulation of CD14-positive monocytes prognosticated decreased survival in renal cell carcinoma patients [38]. There were also elevated CD14-positive monocyte levels in the peripheral blood that correlated with CD14 expression in tumors. Moreover, CD14 was also related to a broad range of ICIs, which implies its potential as a target molecule to enhance the immunotherapy of colorectal cancers [37]. These findings are in line with ours; however, further studies are required to assess the value of plasma sCD14 as a biomarker and estimate cut-off points for its level in plasma samples that could accurately discriminate between responders vs. non-responders or survivors vs. non-survivors.

To sum up, our preliminary study demonstrated several interesting findings: (1) early after the start of the anti-PD-1 therapy (at T_1_), there were the most substantial differences in the circulating cfmDNA composition between patients with distinct clinical outcomes (in terms of the relative abundances of particular phyla, bacterial ASV alpha diversity, and the highest number of differentially abundant genera indicated in the DAA); (2) changes in the circulating cfmDNA composition were also observed at T_1_ in patients with unfavorable clinical outcomes and in those with favorable ones at a later stage of the immunotherapy (at T_n_); (3) baseline circulating cfmDNA signatures correlated with survival outcomes; (4) at T_1_, there was a higher plasma sCD14 level in NR vs. R, while at T_n_, the trend was opposite (also in CB vs. NB comparison)—these trends corresponded with changes in the circulating cfmDNA composition; (5) high baseline sCD14 level and its increase or stability during treatment correlated with poor survival outcomes. These findings suggest the potential value of circulating cfmDNA and plasma sCD14 as predictive and prognostic biomarkers in cancer patients undergoing ICI therapy. However, it should be pointed out that our study revealed an association between analyzed biomarkers and clinical outcomes of the study cohort, but it did not provide mechanistic insight into the link between them and anticancer immune responses. Moreover, many variables that were not examined in our study could potentially affect the results, such as infection, inflammation, and the general clinical condition of patients. For instance, the questionnaire-based analysis indicated that baseline sCD14 level significantly correlated (*p* < 0.05) with the prevailing dietary fat type, probiotic use, and defecation frequency (but not with, e.g., daily consumption of fruit and vegetable or dairy portions that were previously shown to be associated with clinical outcomes [9]) at baseline in the study cohort (data not published). Taken together, our study serves as a foundation for future observational and mechanistic studies that will improve the understanding of the role of circulating cfmDNA and sCD14 in cancer treatment.

Our study has several limitations. Firstly, the study comprised a small number of patients (*n* = 66), which limited the power of statistical analyses. Secondly, we performed V3-V4 16S rRNA gene sequencing to analyze the composition of circulating cfmDNA. This approach is well suited to low-biomass and high-host-DNA samples, such as plasma samples [14]. However, it specifically targets bacterial and archaeal sequences, while viral and fungal sequences, which may also be present in the circulating cfmDNA pool [39], cannot be detected with this method. Zaidi et al. (2022) found that two bacteriophages (i.e., *Salmonella phage jersey* and *Escherichia virus P1* in the serum microbiome) were significantly associated with OS in EAC patients [20]. Moreover, a panel based on those microbes and *E. coli* predicted the OS in those patients. These findings suggest the importance of the detection of all microbial sequences as they may possess a value of prognostic and predictive biomarkers in cancers. Notably, marker gene sequencing results will not reflect bacterial/archaeal sequences that do not cover the amplified region of the targeted gene (e.g., V3-V4 hypervariable region of 16S rRNA gene). Therefore, taxa identified in the plasma samples using such a method may be underrepresented. This may explain, at least partially, some discrepancies observed between studies.

## 4. Materials and Methods

### 4.1. Study Cohort and Clinical Data Collection

Recruitment to the study was conducted in the years 2018–2021 at the Department of Medical and Experimental Oncology, Heliodor Święcicki Clinical Hospital, Poznań University of Medical Sciences (Poznań, Poland). The study cohort comprised patients with histologically confirmed unresectable stage III or stage IV cutaneous melanoma enrolled in treatment with the anti-PD-1 therapy (nivolumab or pembrolizumab) as a part of the Ministry of Health (Poland) drug program (*n* = 66) [40]. All participants signed written informed consent. The study was approved by the Bioethics Committee at Poznań University of Medical Sciences (registration number 402/18).

Clinical information, including tumor stage, serum LDH level, V600E/K mutation in *BRAF* gene, response to the ICI therapy, PFS, and OS, were collected from the medical records. Response to the ICI therapy was assessed according to the response evaluation criteria in solid tumors (RECIST) v.1.1. and patients with complete (CR) or partial (PR) responses were defined as R and those with disease stabilization (SD) or disease progression (PD) as non-responders NR. Additional classification categorized patients into CB, including patients with CR, PR, or SD, and NB, comprising patients with PD. Patients’ categorization into the subgroups at baseline was performed concerning the best overall response they have experienced during treatment. PFS was defined as the time from the anti-PD-1 therapy initiation to the first event, i.e., disease progression or death from any cause. OS was defined as the duration of patient survival from the time of the anti-PD-1 therapy initiation.

### 4.2. Blood Sample Collection and Measurement of Plasma sCD14 Concentration

Blood samples were collected from patients at scheduled time points, i.e., before the anti-PD-1 therapy initiation (T_0_) and during treatment (T_1_ and T_n_, approximately 3 and later than 3 months, mostly about 12 months, from the start of the anti-PD-1 therapy, respectively). Blood was collected in vacutainer tubes containing a separation gel and an anticoagulant (BD Biosciences, Franklin Lakes, NJ, USA). Within one hour of the blood collection, the samples were centrifuged (1500× *g*, 10 min) to separate the blood plasma fraction from the peripheral blood cells. Then, the plasma was transferred to new tubes and centrifuged again (12,000× *g*, 10 min) to pellet the remaining cells and debris. The plasma samples were stored at −80 °C for further analysis.

The concentration of sCD14 in plasma samples (*n* = 152) was measured with the Human CD14 ELISA Kit (Invitrogen, Waltham, MA, USA) following the manufacturer’s protocol.

### 4.3. Circulating cfDNA Extraction and 16S rRNA Gene Sequencing

Circulating cfDNA was extracted from 270 µL of plasma samples (*n* = 73) using MagMAX™ Cell-Free DNA Isolation Kit (Applied Biosystems, Waltham, MA, USA) in MagMAX™ Express (Applied Biosystems), following the protocol of the manufacturer. The instrument was decontaminated with UV light before the cfDNA extraction. The concentration of cfDNA was determined fluorometrically using the QuantiFluor dsDNA System (Promega, Madison, WI, USA) by the instructions of the kit manual.

Subsequently, the library for 16S rRNA gene sequencing was prepared. Firstly, the V3-V4 hypervariable regions of the 16S rRNA gene were amplified. A total of 20 µL of PCR mixture consisted of 2X Platinum SuperFi II PCR Master Mix (Invitrogen), 0.5 µM 341F (5′-TCG TCG GCA GCG TCA GAT GTG TAT AAG AGA CAG CCT ACG GGN GGC WGC AG-3′) and 785R primers (5′-GTC TCG TGG GCT CGG AGA TGT GTA TAA GAG ACA GGA CTA CHV GGG TAT CTA ATC C-3′) [41], and 8 µL of template cfDNA (the concentrations of total cfDNA, host and microbial, were in a range from 0.002 to 0.567 ng·µL^−1^, and the samples were not diluted before amplification). PCR was performed with initial denaturation at 98 °C for 60 s, 25 cycles of denaturation at 98 °C for 10 s, annealing at 55 °C for 15 s, extension at 72 °C for 45 s, and final extension at 72 °C for 10 min. The PCR amplicon concentration was also determined fluorometrically using the QuantiFluor dsDNA System (Promega), and the quality of PCR amplicons was analyzed by agarose gel electrophoresis.

The next steps were performed by Genomed S.A. (Warsaw, Poland). They included the generation of sequencing libraries with NEBNext Ultra DNA Library Prep Kit (New England BioLabs, Ipswich, MA, USA) for Illumina, according to the manufacturer’s recommendations, assessment of library quality with a Qubit 2.0 Fluorometer (Thermo Scientific, Waltham, MA, USA) and Agilent Bioanalyzer 2100 system (Agilent Technologies, Santa Clara, CA, USA), and libraries sequencing on Illumina MiSeq PE300 platform (Illumina, San Diego, CA, USA).

Then, the 16S rRNA gene sequences were processed as described in our previous paper [9]. However, the taxonomy assigned to each merged sequence was performed using the SILVA SSU database release 138.1 with an emended description of the genus *Lactobacillus* Beijerinck 1901 [42]. The list of taxonomic units (1998 ASVs) was reduced for the ‘likely contaminant’ genera (166 ASVs), according to Salter et al. (2014) [43].

### 4.4. Statistical Analysis

The concentration of plasma sCD14, the taxonomic profile of circulating cfmDNA at the phylum level, and bacterial ASV richness (measured by Chao1) and diversity (measured by inverse Simpson, Gini–Simpson, and Shannon indices) of circulating cfmDNA [44] were characterized in the study cohort. The *p*-values describing the statistical significance of differences in the level of plasma sCD14, relative abundance of particular phyla, and bacterial ASV alpha diversity indices between study subgroups were calculated with the *t*-test. The *p*-values ≤ 0.05 were regarded as significant.

Furthermore, DAA was performed using an ANOVA-like Differential Expression version 2 (ALDEx2 v.1.38. R package) tool. The DAA aimed to identify differentially abundant taxa at the genus level for comparisons between R vs. NR and CB vs. NB at every collection time point (to determine differences in the circulating cfmDNA signatures between subgroups with distinct clinical outcomes) and for comparisons between particular collection time points (T_0_ vs. T_1_, T_0_ vs. T_n_, and T_1_ vs. T_n_) within those subgroups (to determine changes in the circulating cfmDNA profiles during anti-PD-1 therapy). The *p*-values describing the statistical significance of DAA results were calculated with the Wilcoxon rank test. The *p*-values < 0.1 were regarded as significant. The direction of changes in the relative abundance of taxa between the two subgroups being compared was determined based on the effect size value. A positive effect size suggests an increase in the relative abundance of a particular taxon in the tested group compared to the reference group, while a negative suggests a decrease. The first group of the two being compared is considered a reference group, whereas the second one is a tested group. Effect size also measures the biological significance of the observed differences (the larger the effect size, the more substantial the difference between subgroups).

The logistic regression was also performed to analyze the effect of the change in plasma sCD14 concentration between the start of the anti-PD-1 therapy (T_0_) and 3 months after its commencement (T_1_) on the treatment effect expressed as R/NR or CB/NB. The *p*-values < 0.1 were regarded as significant.

There was also analyzed an association of the baseline concentration of plasma sCD14, the change in the plasma sCD14 concentration (T_0_ vs. T_1_), and baseline cfmDNA composition with survival outcomes (PFS and OS). Survival analysis and visualization were performed using survival v.3.7. and survminer v.0.5. R packages. PFS and OS rates were plotted with the Kaplan–Meier survival curves, which were compared using the Log-rank (Mantel–Cox) test. The associations between covariates of interest and survival outcomes were examined with the Cox regression. The *p*-values < 0.1 were regarded as significant. The maximally selected rank statistics were used to determine plasma sCD14 concentration and microbial abundance (low vs. high) or plasma sCD14 change (decrease vs. increase/stable). A Cox proportional hazard model was visualized with a forest plot. It shows HR; HR > 1 indicates an increased risk of disease progression and death, while HR < 1, on the other hand, indicates a decreased risk.

All statistical analyses of the data were conducted using R version 4.3.2.

## 5. Conclusions

Our study demonstrated the association between circulating cfmDNA signatures and plasma sCD14 concentration and clinical outcomes in advanced melanoma patients undergoing anti-PD-1 therapy. These findings suggest their potential as predictive and prognostic biomarkers in cancer patients treated with ICIs. Further observational studies on larger cohorts are indispensable to verify the value of these non-invasive and easily accessible biomarkers. Moreover, mechanistic studies are required to explain the source and function of circulating cfmDNA and the association between sCD14 and anti-cancer immune responses. Simultaneous analysis of microbial proteins or epitopes in plasma samples would also provide better insight into the microbial interaction with the host.

## Figures and Tables

**Table 1:** Baseline characteristics of advanced melanoma patients undergoing anti-PD-1 therapy (*n* = 66).

Subject Characteristics	Responders (R; *n* = 28)	Non-Responders (NR; *n* = 38)	*p*-Value (R~NR)	Clinical Benefit (CB; *n* = 39)	No Benefit (NB; *n*= 27)	*p*-Value (CB~NB)
Sex, *n* (%)						
Male	15 (23)	27 (41)	0.197 ^a^	23 (35)	19 (29)	0.438 ^a^
Female	13 (20)	11 (17)		16 (24)	8 (12)	
Age (years),						
median (range)	64 (40–84)	69 (32–92)	0.215 ^b^	63 (32–85)	70 (38–92)	0.084 ^b^
M-stage at diagnosis ^c^, *n* (%)						
IV M1a	7 (11)	9 (14)	0.339 ^a^	13 (20)	3 (5)	0.067 ^a^
IV M1b	5 (8)	5 (8)		7 (11)	3 (5)	
IV M1c	9 (14)	13 (20)		11 (17)	11 (17)	
IV M1d	4 (6)	9 (14)		5 (8)	8 (13)	
IIIc	3 (5)	0 (0)		3 (5)	0 (0)	
Serum LDH, *n* (%)						
Normal (≤250 U/L)	22 (34)	21 (34)	0.111 ^a^	31 (48)	12 (19)	0.014 ^a^
Elevated (>250 U/L)	6 (9)	15 (23)		8 (12)	13 (20)	
Serum LDH						
median (range)	197.5 (121–474)	238 (141–1173)	0.039 ^b^	195 (121–474)	292 (141–1173)	0.003 ^b^
*BRAF* V600E/K mutation, *n* (%)						
Present	9 (14)	16 (25)	0.439 ^a^	13 (20)	12 (19)	0.298 ^a^
Absent	19 (30)	20 (31)		26 (41)	13 (20)	

LDH, lactate dehydrogenase. ^a^ Fisher’s exact test. ^b^ Kruskal–Wallis test. ^c^ American Joint Committee on Cancer (AJCC) 8th edition.

## Data Availability

The data that support the findings will be available in the Repository for Open Data at https://doi.org/10.18150/6F2OSE following an embargo from the date of publication to allow for commercialization of research findings.

## Supplementary Materials

The supporting information can be downloaded at: https://www.mdpi.com/article/10.3390/ijms252312982/s1.
